# Towards an Ideal *In Cell* Hybridization-Based Strategy to Discover Protein Interactomes of Selected RNA Molecules

**DOI:** 10.3390/ijms23020942

**Published:** 2022-01-15

**Authors:** Michele Spiniello, Mark Scalf, Amelia Casamassimi, Ciro Abbondanza, Lloyd M. Smith

**Affiliations:** 1 Department of Precision Medicine, University of Campania Luigi Vanvitelli, 80138 Naples, Italy; ciro.abbondanza@unicampania.it; 2 Division of Immuno-Hematology and Transfusion Medicine, Cardarelli Hospital, 80131 Naples, Italy; 3 Department of Chemistry, University of Wisconsin-Madison, Madison, WI 53706, USA; mascalf@wisc.edu (M.S.); smith@chem.wisc.edu (L.M.S.)

**Keywords:** RNA molecules, ‘*in cell*’ hybridization methods, RNA–protein interactions

## Abstract

RNA-binding proteins are crucial to the function of coding and non-coding RNAs. The disruption of RNA–protein interactions is involved in many different pathological states. Several computational and experimental strategies have been developed to identify protein binders of selected RNA molecules. Amongst these, ‘*in cell*’ hybridization methods represent the gold standard in the field because they are designed to reveal the proteins bound to specific RNAs in a cellular context. Here, we compare the technical features of different *‘in cell*’ hybridization approaches with a focus on their advantages, limitations, and current and potential future applications.

## 1. Introduction

RNA-binding proteins (RBPs) are of primary importance to the structure and function of different RNA species. Messenger RNAs are specifically associated with proteins in dynamic mRNA–protein complexes (RNPs) during their entire cell life cycle [1]. RNA-binding proteins (RBPs) are fundamental for each step of mRNA biology, comprising leading actors in post-transcriptional regulation of gene expression [2]. Additionally, protein interactions with microRNAs (miRNAs) and long non-coding RNAs (lncRNAs) are relevant to the epigenetic, transcriptional and post-transcriptional functions of these non-coding RNAs (ncRNAs) [3,4].

Disruption of ‘physiological’ RNA–protein interactions can cause aberrant mRNA splicing events and/or alteration of ncRNA function, affecting genetic, autoimmune, neurological, and tumoral diseases as well as cancer drug resistance [4,5,6,7,8,9,10,11]. Therefore, targeting of RBPs involved in ‘pathological’ RNA–protein interactions may represent a new therapeutic option in human disease treatment [12,13,14,15,16]. 

Both computational and experimental methods have been developed to study RNA–protein interactions [17,18,19]. Computational tools can predict specific RNA–protein interactions and be used to validate empirical data or to direct functional studies. Experimental strategies reveal the RNAs associated with a given immuno-precipitated protein (protein-centric) or the proteins interacting with a specific targeted RNA (RNA-centric) [20]. In this review, we will focus on experimental methods. For an excellent overview of the computational approaches used to elucidate RNA–protein interactions, see reference [21].

Both protein-centric and RNA-centric experimental approaches are commonly defined as ‘in vivo’ if they reveal the RNA–protein interactions as present in the cell or as ‘in vitro’ if they show an interplay outside the physiological cell context. In this review, we substitute the term ‘in vivo’ with *‘in cell*’ because these methods are applied to cell culture samples that do not adequately represent the complex environment of a living organism. Conversely, vIPR (in vivo Interactions by Pulldown of RNA) can be considered properly an in vivo method because proteins binding to a target RNA are identified from a crosslinked and lysed living system; in this case, *C. elegans* [22].

*In cell* methods identifying the global protein interactomes of selected RNA molecules (RNA-centric) can be divided into the primary categories of tag-mediated/CRISPR-based approaches and hybridization capture approaches. In the tag-mediated approach, the target tagged RNA construct is transfected and expressed in a specific cell system and the RNA–protein complexes formed are purified through the associated tag. Examples of tag-mediated methods are: tandem RNA affinity purification (TRAP) [23], RNA affinity in tandem (RAT) [24], RNA–protein interaction detection (RaPID) [25], RBP purification and identification (RaPID) [26,27], MS2 biotin tagged RNA affinity purification (MS2-bioTRAP) [28], tobramycin-based tandem RNA isolation procedure (tobTRIP) [29] and similar methods [30,31,32]. Recently, a CRISPR-based approach, named CRISPR-based RNA-United Interacting System (CRUIS), provides the tracking of the target RNA, the editing of specific sequences into the transcript and, through the PafA enzyme fused to dCas13a, the linking to the surrounding RNA-binding proteins [33]. Despite their flexibility, a drawback of these methods is that the alteration of the native RNA tridimensional structure in tag- and CRISPR-based approaches may cause RNA–protein interaction artifacts and/or the loss of the true interactors [2]. 

Hybridization approaches are able to identify RBPs associated with specific transcripts reflecting the biological cell environment because they are based on purification of the endogenous target RNA that has been cross-linked with the associated proteins [2,34]. Several *in cell* hybridization methods have been developed: ChIRP-MS (comprehensive identification of RNA binding proteins by mass spectrometry), CHART-MS (capture hybridization analysis of RNA targets and mass spectrometry), RAP-MS (RNA antisense purification and mass spectrometry), HyPR-MS (hybridization purification of RNA–protein complexes followed by mass spectrometry), and PAIR (peptide nucleic acid (PNA)-assisted identification of RBPs). Other similar approaches have been applied to specific biological questions [35,36]. 

The ideal *in cell* RNA-centric hybridization method should be easy to set up and to perform in terms of cost and time, should guarantee a high efficiency and specificity, and should be versatile enough to function in different biological environments. Here, based on these parameters, we compare five recently developed hybridization approaches in terms of their user-friendliness, purification, and post-capture phases. We also discuss possible future directions of these methods.

## 2. Experimental Design

### 2.1. Capture Oligonucleotide Design

Capture Oligonucleotide (CO) design represents the first critical step that impacts on the success of the entire experiment. The ideal COs should hybridize to the target RNA with high specificity and efficiency. CO number, length, composition, modification, and the choice of the target RNA region are tightly interconnected with the different capture strategies and hybridization conditions (see Table 1 and Section 3.3 for more details).

**Figure 1:**
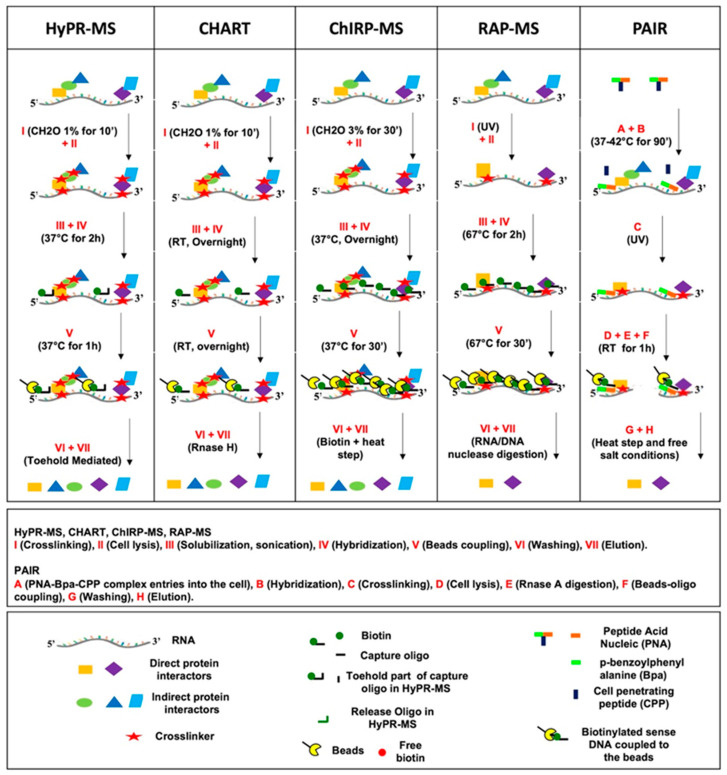
Schematic representation of in cell hybridization-based methods workflow [37,38,39,40,42,43,44,45,46].

Almost all the designed COs are 20–30 nucleotides long (although 90 nt in RAP-MS), consist of unmodified DNA or RNA sequences, and are biotinylated in order to permit the isolation of the hybridized RNA–protein complexes with streptavidin-coated magnetic beads (Table 1). Differently from the other methods, the COs in PAIR consist of a PNA (peptide nucleic acid) part coupled to the cell-penetrating peptide transportan 10 (TP10), and to p-benzoylphenylalanine (Bpa), a photoactivatable compound (Figure 1). The PNA hybridizes to the target RNA after its delivery to the cell through TP10 (Figure 1) and subsequent UV irradiation induces crosslinking of the RNA-interacting molecules through the reactive Bpa. Biotinylated DNA ‘sense’ oligos coupled to streptavidin magnetic beads serve to isolate the hybridized PNA–RNA–protein complexes (Figure 1). PNA ‘COs’ are highly specific and create stable and protease/nuclease resistant hybrids [41], but their synthesis is expensive and time-consuming.

Focusing heavily on the fundamental aspect of probe design, CHART utilizes an RNAse H assay to identify the accessible single-stranded regions, under the same crosslinking conditions as in the capture experiment. This approach is highly accurate but expensive and time-consuming due to the high number of probes and qPCR assays needed to perform the RNAse H assays (Table 2). The best COs from the pool obtained from the RNAse H assays are empirically determined and chosen for the capture experiment [37,38]. Similarly, in the PAIR method, RNA regions available for CO annealing are chosen by means of an in-situ hybridization screening [41]. CHIRP and RAP bypass this step by using a tiling strategy, performing the RNA purification independently of knowledge of the RNA single-stranded regions. This tiling approach provides faster results, but it is expensive because of the use of a high number of COs to cover the entire RNA length (Table 2) [39,40]. HyPR-MS performs an in-silico analysis using the freely available software (Mfold database) [47] to predict single-stranded regions of the target RNA. Mini-scale experiments using the in silico designed COs permit empirical determination of the best COs [42,43,44,45,46]. This approach is advantageous in terms of both time and cost (Table 2).

### 2.2. Cell Number and Cell Type Choices

Cell number and cell type choices should take into account both technical and biological aspects. Ideally, the RNA to be studied should be analyzed in a cell system where it is expressed at high levels. At the same time, a cell line should be chosen where the selective RNA–protein interactome identification to be investigated supports the biological motivation for the study. Theoretically, the lower the RNA abundance in the chosen cell type, the greater the number of cells that will need to be employed. Table 1 reports number and types of cells used per proteomic experiment for each hybridization method and the estimated copy number per cell of the studied RNA, if reported in the published manuscript. The protein interactome of abundant transcripts (>1000 copies per cell), such as the U1 and U2 snRNAs, and the Xist, Malat, and Neat lncRNAs, have been successfully identified using around 1–8 × 10^8^ cells. On the other hand, the discovery of the protein interactors of c-Myc (60 copies per cell) [46] and *Ankh* mRNAs [41], of Norad (380 copies per cell) [45], SAMMSON [35], and SPRY4-IT1 lncRNAs [36], still using a ‘reasonable’ number of cells (1–5 × 10^4^–1 × 10^8^ cells), shows the efficacy of *in cell* hybridization methods for less abundant targets. Increasing the cell number represents the most readily accessible experimental parameter to isolate an adequate number of capture-eluted transcripts and consequently of the associated proteins in order to be above the limit of detection of the mass spectrometer. In reality, selecting a cell system expressing high levels of the studied transcript while also obtaining high capture efficiency and mass spectrometry sensitivity is not always straightforward. On the other hand, growing a large number of cells can increase costs and potentially increase non-specific background. Therefore, it is important to choose a system that results in a ‘sweet spot’ for the number of cells needed. By considering mass spectrometry sensitivity for peptides in complex biological samples (~low femtomoles; 1 fmol = 10^–15^ mol = 6 × 10^8^ molecules) [48] and measuring the starting amount of the target transcript and its captured/eluted fraction, it is possible to estimate the approximate number of cells to use per experiment, as shown in several HyPR-MS manuscripts [44,45,46].

Finally, the use of a ‘comparative’ strategy, either using different cell types or conditions that serve to highlight differences, may help in identification of RBPs with specific biological roles. For example, an impressive ChIRP study [40] used three cell types harboring four Xist ‘states’ (‘turned off’, ‘turned on’, ‘random’ expression, and ‘silenced’) revealing a specific pattern of RBPs related to different cell differentiation states. In the same study, full-length Xist and A-repeat mutant comparison allowed the detection of proteins binding selectively to the deleted domain [40]. In another study conducted using PAIR [41], interesting variations in Ankh mRNA protein interactors under different cell conditions (‘basal’, BDNF, 5-dihydroxyphenylglycine (DHPG) stimulation, or high potassium treatment) were observed, showing how specific external stimuli can impact dynamic mRNP remodeling [41]. Despite such examples, further improvements in ‘comparative’ strategies are necessary, as discussed in the future perspectives paragraph.

## 3. Purification Procedure

Crosslinking treatment, cell lysis and lysate preparation for hybridization, target RNA capture, bead coupling, washing, and elution steps are all common steps in almost all *in cell* hybridization protocols (Figure 1) and each specific technical choice can impact on each method’s cost, time, versatility, efficiency, and specificity, as reported in Table 3 and analyzed below.

### 3.1. Crosslinking

As regard to the crosslinking (Figure 1; I, A), UV and formaldehyde are the cross-linkers used in RNA hybridization methods (Table 1) to covalently ‘freeze’ RNA–protein interactions.

UV irradiation with 254 nm ultraviolet light excites RNA/DNA nucleobases that then react with amino acids in close proximity [49,50]. UV crosslinking efficiency is generally low (1–5%) and misses direct RBP interactions with structures other than the nucleobases, generating a potential high rate of false negatives [2]. Furthermore, some nucleotides and amino acid residues as well as selected RNA structures are more amenable to UV crosslinking than others, creating a systematic bias. Specifically, pyrimidines are more photoactivatable than purines; Cys, Lys, Phe, Trp, and Tyr residues have the highest UV crosslinking efficiencies amongst amino acids; and proteins bound to single-stranded regions of RNAs are more efficiently crosslinked than those bound to double-stranded regions [51,52]. Based on the mechanism and characteristics of UV crosslinking, RAP identifies direct RBPs with high specificity for true interactors (crosslinking at zero distance) but with a potential high rate of false negatives, given the low and non-uniform crosslinking efficiency. Furthermore, indirect protein binders are missed, which comprise an important part of the protein ‘cloth’ of RNA molecules [51,53]. Differing from RAP, PAIR uses a photoactivatable compound, p-benzoylphenylalanine (Bpa), included into the capture probe system, that, after UV irradiation, is activated and crosslinks the RBPs associated with the hybridized target RNA (Figure 1). PAIR, in the same way as RAP, identifies only direct RNA protein binders given the short action range (4.5 Å or less) of the activated Bpa [41]. Finally, UV irradiation may produce RNA chain breaks [54] with the potential loss of capturing these target RNA sequences in approaches such as PAIR that do not use full tiling (Table 1, and see Section 3.3).

Formaldehyde, a crosslinking agent able to cross both cell and nuclear membranes, reacts with nucleophilic groups on amino acids or on DNA/RNA bases forming methylol intermediates in a first step. Successively, these methylol adducts can be converted into Schiff bases capable to react with other nucleophilic groups present on proteins or nucleic acids, creating a variety of crosslinked products [55]. The use of formaldehyde crosslinking in CHART-MS, ChIRP-MS, and HyPR-MS allows the discovery of the entire protein composition of ribonucleoprotein complexes, but without the possibility to differentiate between direct and indirect interactors [50]. However, bioinformatic software and publicly available databases may help to differentiate between direct and indirect binders, as in Hy-PRMS [46]. Furthermore, the reversibility of formaldehyde-mediated bonds, under relatively mild heating conditions in an appropriate buffer, facilitates the recovery of proteins for mass spectrometry analyses [18,55,56]. Conversely, extensive formaldehyde treatment may crosslink non-specific interactors [34] and induce breaking of target RNA [57] with the potential loss of these as capturable target RNA sequences in approaches such as CHART-MS and HyPR-MS that do not use full-tiling. Milder formaldehyde crosslinking conditions in HyPR-MS (1% for 10 min) compared to those in CHART-MS and ChIRP-MS (Table 1), combined with a multiple probe capture strategy (see below), allows for high purification specificity while still maintaining a robust hybridization rate along all RNA target sequences (Table 3) with an adequate protein recovery (Table 4) [45].

### 3.2. Cell Lysis and Lysate Preparation for Hybridization

With the exception of PAIR, after chemical cell lysis, the crosslinked lysate is sonicated and/or digested in order to permit its solubilization before hybridization (Figure 1; II–III). The median size of the fragmented RNA ranges from 150 bp to 6 kb amongst the different methods (Table 1). Milder solubilization conditions are preferred given the potential breakage of target RNA strands with the potential loss of these as capturable RNA sequences in those approaches not employing a full tiling capture strategy.

### 3.3. Hybridization

Hybridization represents the core of the procedure, and its success depends on an interconnected set of parameters related to the hybridization conditions and to the capture strategy (Figure 1; IV, B). 

Salt and denaturant concentrations, pH conditions, CO sequence composition, modifications, length, and concentration are well defined variables in hybridization procedures, impacting the melting temperature (Tm) of hybridization. Usually, hybridization temperature (Thy) should be set to 18–24 °C below the calculated CO Tm (perfect hybrids) to achieve a greater hybridization rate [58,59]. Denaturants, such as urea and formamide, decrease nucleic acid Tm and denature DNA or RNA molecules yielding single stranded regions for probe annealing [60,61]. Salt and denaturant concentrations have been chosen in RAP-MS, CHIRP-MS and CHART-MS protocols to yield efficient RNA hybridization (Table 1 and Table 3). In contrast, no denaturing agents are used in PAIR and HyPR-MS (Table 1). The presence of single stranded regions in the structure of RNA molecules, their prediction, targeting, and empirical confirmation as in HyPR-MS, permit a successful hybridization, obviating the need for any destabilizing molecules [42,45,46]. 

Based on the number of COs employed to study a target RNA, *in cell* hybridization methods can be divided into full tiling and single/multiple probe strategies. RAP-MS and ChIRP-MS use COs covering the target RNA along its full length (full tiling strategies), avoiding the potential loss of fragments created by crosslinking and/or solubilization steps and permitting a homogeneous and robust capture (Table 1 and Table 3). However, the use of a high number of COs could negatively affect specificity and does not permit the discrimination of multiple splice isoforms derived from the same gene. This point is particularly relevant if we consider that aberrant transcript variants involved in tumorigenesis and cancer drug resistance often differ from their normal counterparts only by short sequences [8]. On the other hand, the single/multiple probe approaches have greater versatility and theoretical specificity (Table 3) but may miss specific RNA fragments that are not targeted by the COs chosen. This multiprobe system, along with the absence of a sonication step, reduces the possibility of missing fragments of target RNA, although it remains possible given the UV irradiation (Table 2). Specifically, ChIRP-MS and PAIR use, respectively, two and three COs against different RNA regions and both methods require separate captures for each CO used [41]. HyPR-MS uses two or three COs distributed uniformly along the linear structure of the target RNA in one single capture step, reducing cost and showing adequate values of capture specificity and efficiency in different regions of the studied transcript (Table 1 and Table 3) [45,46]. 

### 3.4. Bead Coupling and Washing

After hybridization of target RNA with the crosslinked proteins, RBP complexes are captured by streptavidin-conjugated magnetic beads and washed to remove nonspecific interactors (Figure 1; V–VI). Stringent washing conditions, consisting of higher washing temperature (Tw) and lower salt concentration than those used during hybridization, are recommended to improve specificity [58]. All methods use a washing temperature and salt concentration similar to the corresponding hybridization step, with the exception of CHART-MS and ChIRP-MS employing milder rinsing salt conditions, (Table 1).

### 3.5. Elution

Different elution strategies have been developed to release RNA interacting proteins from beads (Figure 1; VII). CHART-MS and RAP-MS use an enzymatic strategy, digesting respectively only the RNA of the hybrid between the target RNA and the DNA probe [38] or both RNA/DNA molecules using a benzonase nuclease enzyme (Table 1) [39]. ChIRP-MS uses a gentle biotin elution coupled to a heat step in order to release biotinylated oligo from streptavidin beads and to de-crosslink RNA associated proteins [40]. On the other hand, the high temperature and salt-free conditions in PAIR allow denaturation of the triplex hybrid comprised of biotinylated DNA, PNA, and target RNA with the crosslinked proteins [41]. Finally, HyPR-MS uses release oligonucleotides (RO) that are fully complementary to the corresponding Cos (either to the hybridization sequence or to the toehold release sequence), displacing the target RNA, which is eluted together with the crosslinked proteins. This toehold strategy permits the purification of multiple transcripts from the same cell sample by sequentially adding specific Ros for different targets previously hybridized, thereby reducing cost and time requirements and permitting comparison of different RNAs or normal and aberrant splice variants, and avoiding differences caused by technical variability (Table 1 and Table 3) [42,45,46].

## 4. Post-Purification

After elution, RNA-interacting proteins are purified and trypsin digested before identification using mass spectrometry. The use of proteomic quantification approaches, such as isotope labeling in RAP-MS [39] and label-free quantification in CHART-MS and HyPR-MS [38,42,45,46] facilitate differentiation of valid from false interactors [62]. Specifically, robust cut-off values and inclusion criteria can be established by using more technical replicates and by comparing the proteome of the target RNA against those from one or more controls. Different control types have been used such as lysate (input) or protein interactomes obtained by capture probes directed against RNAs different from the target or against nothing (scrambled probe, hybridization in cells not expressing the studied transcript or after RNase treatment) (Table 1) [38,39,42,56]. Hypothetically, the more controls that are employed the more accurate are the results of data analysis, but at the cost of increased time and expense. Furthermore, for an ideal comparison, the target RNA and the control should be studied from the same cell type and under the same conditions. HyPR-MS addresses these issues through its multiplexing power, using multiple controls (scrambled, poly-dT COs) from the same cell preparation, thereby reducing time, cost, and potential technical variability. 

Literature analysis, bioinformatics tools, such as Gene Ontology Enrichment Analysis [63], STRING database [64], BioGRID [65], CORUM complex [66], PrePPI [67] Pfam [68], and UniProt database [69], can help to functionally organize and interpret the results. 

RNA protein interactome validation studies are performed using various strategies as listed in Table 4. This step measures the capacity of methods to identify true protein interactors, reflecting both purification and post-purification phases. In summary, all of the *in cell* hybridization approaches are reliable in identifying protein interactomes of selected RNA molecules (Table 4).

Finally, functional studies can be performed for the most interesting RBPs to discover the biological role and relevance of each interaction. A reliable strategy to reveal the functional link between the target RNA and the identified RBP is the knockdown of the protein interactor and subsequent assessment of studied transcript changes measuring: (a) transcript levels (using RT-qPCR), (b) translated protein levels (using Western Blot; only if it is studied a coding RNA), and (c) transcript function of non-coding RNAs or biological processes accomplished by the product of the target coding transcript (using a specific assay related to the biological process in which is involved the studied non coding RNA or the translated protein). Examples of this approach include specific Xist interactors identified in RAP-MS and ChIRP-MS that affect lncRNA mediated silencing [39,40], or of selected HIV-1 RNA splice variants binders discovered using HyPR-MS and involved in virus replication regulation [42,43].

## 5. Conclusions and Future Perspectives

Current *in cell* hybridization methods have shown accuracy in identifying protein interactomes of selected RNA molecules (Table 4); however, their widespread adoption is still limited. PAIR was the first in cell hybridization method introduced [41], followed by CHART-MS [37,38], ChIRP-MS [39], RAP-MS [40], and HyPR-MS [42]. HyPR-MS, developed most recently, provides some advantages with respect to the factors of cost, time, technical accessibility, accuracy, and versatility (Table 1 and Table 3). Each method includes intrinsic limitations and advantages and there is no one approach superior to the others in all aspects (Table 1 and Table 3). Therefore, implementation of the existing *in cell* hybridization methods and development of new ones should be an ongoing process, attentive to technological innovations and new biological methodologies and at the same time linked to past experience. 

The main challenge for the widespread adoption of in vivo hybridization methods is the study of low copy number RNAs (<50 copies per cell), which represent the most abundant transcripts [70], decreasing cell number currently used per experiment (~10^8^ cells) and, consequently, time and economic requirements. The improvement of capture efficiency and/or mass spectrometry (MS) sensitivity becomes primary for this issue. In this context, the further expansion of multiplex capabilities of HyPR-MS, past the three-fold multiplex capability, and MS based proteomics technological advances, like single-cell proteomics, could allow for a scaling down of material. 

Furthermore, although most of RNA species, like mRNA, lncRNA, small nuclear RNA (snRNA), viral RNA, and rRNA, have been studied using in vivo hybridization methods, it is yet to be defined if tRNA or miRNA are accessible for hybridization purification.

New biological approaches should be considered to improve the current state of the art. For example, cell cycle synchronization and transcript activation or silencing may be performed to have a more controlled experiment with reduced potential noise arising from the cell cycle and functional state heterogeneity. In ChIRP-MS, this issue has been partially addressed by engineering a cell system with a doxycycline-inducible Xist cDNA [40].

Another issue that should be further explored is related to the comparison of the RNA–protein interactomes. Several conditions can be compared (the same transcript in different cell conditions or types, different RNAs, or splice variants of the same RNA in the same cell system) and each one can be useful for a specific purpose. Currently, the information obtained by a ‘comparative’ strategy is focused on the protein interactors differing between the two tested conditions. For a more in-depth analysis, quantitative and post-translational modification (PTM) differences of the shared proteins should be considered. In fact, it is possible that the same RNA protein interactor found in both conditions could exhibit a distinct biological function if present at a different level or with a different PTM pattern.

Finally, another challenge could be the application of *in cell* hybridization methods to tissue samples (*in tissue* hybridization methods) to obtain information more pertinent to the reality of complex living organisms. 

## Figures and Tables

**Table 1:** Experimental conditions used in *‘in cell*’ hybridization methods to identify proteins associated to selected RNAs.

PROCEDURE	CHART-MS [37,38]	ChIRP-MS [39]	RAP-MS [40]	PAIR [41]	HyPR-MS [42,43,44,45,46]
Type, cell amount used and target RNA studied with copy number per cell (cpc) estimation	MCF-7 and BJ cells (10^8^ cells) - Neat lncRNA - Malat-001 lncRNA	HeLa S3 cells (1–5 × 10^8^ cells) - U1 snRNA (~1 × 10^6^ cpc) - U2 snRNA - 7 SK snRNA (2 × 10^5^ cpc) ESC, EpiSC, and TSCs cells (1–5 × 10^8^ cells) - Xist lncRNA (<2000 cpc) ES cells - A-repeat mutant Xist	- Wild-type V6.5 ES cells (2–8 × 10^8^ cells) - U1 snRNA - 18S rRNA - pSM33 cells (2–8 × 10^8^ cells) - Xist lncRNA	Cortical cells (1–5 × 10^4^) - ank mRNA	HIV-1 infected Jurkat cells (5–7.5 × 10^7^ cells) - unspliced full length HIV-1 RNA - HIV-1 RNA splice variants PC3 cells (1 × 10^8^ cells) - Neat lncRNA (1000 cpc) - Norad lncRNA (380 cpc) - Malat-001 lncRNA (3800 cpc) k562 cells (1 × 10^8^ cells) - C-Myc mRNA (60 cpc)
Controls	Lysate	Xist lncRNA capture: - Lysate - U1 snRNA - U2 snRNA - 7 SK snRNA - Non-targeting probe - Samples RNase treated	- Xist lncRNA capture: - Xist capture in cells not expressing Xist - Xist capture in cells not crosslinked - 45 pre-ribosomal RNA	- GluR2mRNA - Lysate - No irradiation - No PNA - No irradiation, no PNA	- Lysate - Poly(dT) - Scrambled
Crosslinking	1% formaldehyde for 10 min on cells and 3% formaldehyde for 30 min on nuclei	3% formaldehyde for 30 min	UV irradiation	UV irradiation of Bpa, a photoactivatable compound linked to capture probe system	1% formaldehyde for 10 min (0.25% formaldehyde for 10 min in HIV1 splice variant study)
Solubilization	Sonication; 3 kbp is median size of chromatin fragments	Sonication; 100–500 bp is the length of chromatin fragments	Sonication and DNAse digestion;100–300 bp is the size of chromatin fragments length		Sonication; About 6 kbp is the median size of chromatin fragments
Probes features	25 nt DNA biotinylated at 3′ Tm 55–65 °C	20 nt DNA biotinylated at 3′	90 nt DNA biotinylated at 3′	PNA coupled to a CPP and to a Bpa Biotinylated sense DNA (antisense to PNA)	Hybridization part of 20–30 nt DNA biotinylated at 3′ and toehold part of 8 nt DNA Tm 56.8–68 °C
Hybridization	2 COs per target each one used for a single experiment 1.3 M urea, 800 mM NaCl, 33 mM HEPES pH 7.5, 0.33% SDS Overnight at 20 °C	43 COs per Xist lncRNA (1 probe/100 bp of RNA length) 15% formamide, 750 mM NaCl, 1% SDS, 50 mM Tris-Cl pH 7.0 Overnight at 37 °C	142 COs per Xist lncRNA (probes span the entire length of the target RNA) 4 M urea, 500 m MLiCl, 10 mM Tris pH 7.5, 0.2% SDS 67 °C for 2 h	3 COs (PNA) per target each one used for a single experiment - 25 mM Hepes pH 7.4, 0.75 mM Na_2_HPO_4_, 70 mM NaCl - 90 min in cell culture at 37–42 °C	2–3 COs per target per experiment 375 mM LiCl, 50 mM Tris pH 7.5, 1% LiDS 37 °C for 2 h
Beads coupling	Overnight, RT	37 °C for 30 min	67 °C for 30 min	1 h at RT (Biotinylated sense DNA coupled to the beads)	37 °C for 1 h
Washing	250 mM NaCl, 0.22% SDS, 10 mM Hepes pH 7.5 Five washing cycles at 20 °C (RT)	300 mM NaCl and 30 mM Sodium citrate (2X SSC), 0.5% SDS Five washing cycles at 37 °C, each one of 5 min	4 M urea, 500 mM LiCl, 10 mM Tris pH 7.5, 0.2% SDS Sic washing cycles at 67 °C for 5 min	25 mM Hepes, pH 7.4, 0.1% Triton X-100, 300 mM NaCl Two washing steps at RT	375 mM LiCl, 50 mM Tris pH 7.5, 0.2% LiDS, 0.2% Triton X-100 37 °C for 15 min
Elution	RNase-H digestion	Biotin-elution at RT for 20 min and at 65 °C for 10 min	Benzonase nonspecific RNA/DNA nuclease digestion for 2 h at 37 °C	50 °C for 20 min in salt-free buffer	Toehold mediated release (RT for 30 min)

Bpa (p-benzoylphenylalanine); PNA (peptide nucleic acid); and CPP cell penetrating peptide.

**Table 2:** Comparison amongst in cell hybridization methods based on setting-up features that impact their wide-spread diffusion.

		CHART-MS	ChIRP-MS	RAP-MS	PAIR	HyPR-MS
SETTING-UP	Cost saving	•	••	••	•	•••
	Time saving	•	•••	•••	•	••
	Technical accessibility	•••	••	••	•••	••
	Characterization level	••	•••	•••	••	••

One circle represents least desirable, two circles represent average, and three circles represent most desirable.

**Table 3:** Comparison amongst in cell hybridization methods based on procedure features that impact their wide-spread diffusion.

		CHART-MS	ChIRP-MS	RAP-MS	PAIR	HyPR-MS
PROCEDURE	Cost saving	••	••	••	••	•••
	Time saving	••	••	••	••	•••
	Versatility	••	•	•	••	•••
	Purification Efficiency	• MALAT, NEAT lncRNAS (1–10%) *	••• XIST lncRNA (>60%) *	••• XIST lncRNA (>60%) *		•• UFL HIV-1 RNA (35%) * US, PS, CS HIV-1 RNA (>70%) ** MALAT lncRNA (8%) * NEAT lncRNA (20%) * NORAD lncRNA (28%) * C-Myc mRNA (30%) *
	Purification Specificity	58S RNA enrichment using NEAT and MALAT COs (<1%) ## NEAT, MALAT DNA enrichment (<1%)	No GAPDH detected using XIST Cos ##	18S RNA enrichment using XIST COs (~1%) ##		UFL HIV-1 RNA (60-fold) # US, PS, CS HIV-1 RNA (10-fold comparing one HIV splice variant to the other two or at least 200-fold relative to cellular RNAs) # - MALAT, NEAT, NORAD lncRNAs (10–110-fold) # - MALAT, NEAT, NORAD DNA capture (<0.1%) - C-Myc mRNA (45-fold) # - C-Myc DNA capture (<0.03%)

One circle represents least desirable, two circles represent average, and three circles represent most desirable. * Purification efficiency is calculated by measuring the amount of RNA target captured compared to the amount of total target in the initial lysate. ** Purification efficiency is calculated by measuring the amount of each HIV splice variant class in the lysate before (pre-lys) and after (post-lys) the capture. Purification specificity is calculated by measuring the amount of a given RNA target captured (using the complementary COs), divided by the amount of that same RNA captured using the COs for different RNAs or for the scrambled CO (#) or measuring the amount of off-target RNA captured using the CO for a specific target (##). In the first case (#) higher values are associated with a greater specificity and vice versa in the second solution (##).

**Table 4:** RNA protein interactome validation strategies in ‘*in cell*’ hybridization methods [37,38,39,40,41,42,43,45,46].

		CHART-MS		ChIRP-MS			RAP-MS			PAIR	HyPR-MS					
STUDIED RNA (lncRNA, snRNA, rRNA, mRNA, HIV-1 RNA)		*Malat001*	*Neat*	*U1*	*U2*	*Xist*	*U1*	18S	*Xist*	*ANK*	*U*S full-length *HIV-1*	*US*, *PS*, *CS* *HIV-1*	*Malat-001*	*Neat*	*Norad*	*c-Myc*
IDENTIFIED PROTEINS		69	71	418	370	81	9	105	10	13	189	926 (CI) 212 (DI)	127	94	415	229
V A L I D A T I O N	Previous known interactors						9/9				92	117/926 49/212	52	24	33	25
	Interacting proteins ‘functionally’ linked to the related RNA	~42	~41	143	143	13		98	9	13	89	633/926 131/212	~117	~66	145	209
	Direct validation	2/69–2/71 (WB)		1 (IP)		3 (IB)			8 (IP)	2 (IP)	8 (siRNA KD and FM)	84 (DI) (siRNA KD) 15 (DI) (siRNA KD and IB)	2/127–2/94–2/415 (IP)			2 (IP)

US (Unspliced) (US); PS (Partially Spliced); CS (Completely Spliced); CI (Common Interactors); DI (Differential Interactors); WB (Western Blot); IP (Immunoprecipitation); IB (Immunoblot); KD (Knockdown); and FM (fluorescence microscopy).
